# Data on clinical characteristics of a heart failure patients’ cohort with reduced ejection fraction and analysis of the circulating values of five different heart failure biomarkers; high sensitivity troponin T, galectin-3, C-terminal propeptide of type I procollagen, soluble AXL and BNP^☆^

**DOI:** 10.1016/j.dib.2016.10.020

**Published:** 2016-11-03

**Authors:** M. Batlle, B. Campos, M. Farrero, M. Cardona, B. González, M.A. Castel, J. Ortiz, E. Roig, M.J. Pulgarín, J. Ramírez, J.L. Bedini, M. Sabaté, P. García de Frutos, F. Pérez-Villa

**Affiliations:** a Institute of Biomedical Research August Pi i Sunyer (IDIBAPS) and the Cardiovascular Clinic Institute, Hospital Clínic de Barcelona, Spain; b Department of Public Health, Universitat de Barcelona, Spain; c Heart Failure and Transplant Unit, Cardiovascular Clinic Institute, Hospital Clínic de Barcelona and researcher at Institute of Biomedical Research August Pi i Sunyer (IDIBAPS), Barcelona, Spain; d Core Laboratory, Hospital Clínic de Barcelona, Spain; e Heart Failure Unit at the Cardiology Department, Hospital de la Santa Creu i Sant Pau, Institut de Recerca Biomèdica (IIB Sant Pau), Universitat Autònoma de Barcelona, Barcelona, Spain; f Pathological Anatomy Department, Hospital Clínic de Barcelona, Spain; g Department of Cell Death and Proliferation at Institut d´Investigacions Biomèdiques de Barcelona (IIBB-CSIC) and IDIBAPS, Barcelona, Spain

**Keywords:** Heart failure, High sensitivity troponin T, Galectin-3, C-terminal propeptide of type I procollagen, AXL receptor tyrosine kinase, Brain Natriuretic Peptide

## Abstract

In this article, the full description of a heart failure with reduced ejection fraction (HF_REF) cohort of 192 patients is provided. Tables with the baseline demographic, prior history, ECG parameters, echocardiographic parameters, laboratory values and pharmacological treatment of these patients are included. Also, the quartile values of the analyzed circulating biomarkers: high sensitivity Troponin T (hs-TnT), galectin-3 (Gal-3), C-terminal propeptide of type I procollagen (CICP), soluble AXL (sAXL) and Brain Natriuretic Peptide (BNP) are given. The main demographic and clinical features of the patients’ subgroups that have hs-TnT, Gal-3, CICP or BNP above the third quartile are described. Tables with Pearson correlation analysis of the HF_REF patients’ biomarker levels are included. And Pearson correlation analysis of the HF_REF patients’ hs-TnT, Gal-3, CICP levels with patients’ biochemical parameters, blood count and inflammation parameters are also described. These data are related to the research articles (AXL receptor tyrosine kinase is increased in patients with heart failure (M. Batlle, P. Recarte-Pelz, E. Roig, M.A. Castel, M. Cardona, M. Farrero, et al., 2014) [1] and Use of serum levels of high sensitivity troponin T, galectin-3 and C-terminal propeptide of type I procollagen at long term follow-up in Heart Failure patients with reduced ejection fraction: comparison with soluble AXL and BNP (M. Batlle, B. Campos, M. Farrero, M. Cardona, B. González, M.A. Castel, et al., 2016) [2].

**Specifications Table**

**Table:** 

Subject area	*Biomedicine*
More specific subject area	*Cardiology, Heart Failure*
Type of data	*Tables*
How data was acquired	*A visit in two specialized outpatient Heart Failure units as well as retrieval of historical records. The circulating biomarker levels were quantified from serum and plasma collected from the patients on the enrolment day.*
Data format	*Filtered, analyzed.*
Experimental factors	*Blood samples from the HF patients were collected on from an antecubital vein. To measure BNP levels, whole blood was collected in a chilled tube with the anticoagulant EDTA and was centrifuged at RCF 1800 g for 10 min at 4 °C. Serum samples for hs-TnT, Gal-3, CICP and sAXL analysis were kept at room temperature for at least 30 min after blood extraction and were later centrifuged at RCF 1800 g for 10 min at room temperature. The supernatants were collected, aliquoted and kept at −80 °C until analysis.*
Experimental features	*The ELISA assays used to quantify the circulating biomarkers’ levels were:* – *the high sensitivity TnT assay (Troponin T high sensitive (05092744 119), Roche Diagnostics)* – *Galectin-3 Platinum ELISA (BMS279/2CE, eBioscience)* – *the MicroValue CICP ELISA assay (8003, Siemens Diagnostics).* – *a chemiluminometric immunoassay run on the ADVIA Centaur Immunochemistry analyzer for BNP (Siemens Diagnostics).* – *sAXL quantification was devised in our laboratory using commercially available antibodies.*
Data source location	*Hospital Clinic and Hospital Sant Pau, Barcelona, Spain.*
Data accessibility	*The available data is with this article*

**Value of the data**

•The data presented in this DIB article is important for interpretation of the circulating levels of the biomarkers that we describe in our study.

•Heart failure is the end-stage of many heart diseases and its development can be highly variable among patients.

•Many results from the literature are difficult to compare due to heterogeneity of the heart failure cohorts analyzed.

•The full description of the HF cohort patients and of the circulating biomarkers and their relationship with the clinical characteristics of the patients will provide deeper insight to the researchers that work in the same field and will allow more meaningful comparisons.

## 1 Data

In this Data in Brief article, we provide the baseline demographic, prior history, ECG, echocardiographic, laboratory and pharmacological parameters of 192 patients with heart failure and reduced ejection fraction (Table 1). The distribution of the circulating values of five biomarkers and their relationship with the patient´s clinical characteristics is also given Table 1, Table 2, Table 3, Table 4, Table 5, Table 6.

## 2 Experimental design, materials and methods

### 2.1 Patient enrolment and collection of clinical data

A detailed description of subject enrollment and collection of clinical data has been reported previously [1].

### 2.2 Data analysis

Descriptive values are given as mean and standard error of the mean (SEM), or as frequencies (%) or as quartile values. Correlation analysis among biomarkers and these with clinical laboratory values were performed with Pearson correlation coefficient. Statistical analysis was performed using the SPSS software. Statistical significance was indicated by *P* value <0.05. A detailed description data analysis can be found elsewhere [1], [2].

## Figures and Tables

**Table 1:** Clinical characteristics of HF patients.

Parameter	Mean±SEM or %	*n*
**Demographics**		
Age (years)	62±1	192
Male (%)	85	164
Female (%)	15	28

**Risk factors**		
Hypertension (%)	76	143
Dyslipidemia (%)	62	115
Diabetes mellitus (%)	35	66
Current/former smoker (%)	70	132
Previous AMI (%)	48	90

**Etiology**		
Idiopathic (%)	28	53
Ischemic (%)	48	93
Valvular (%)	12	23
Hypertensive (%)	2.6	5
Other (%)	9.4	18

**Clinical characteristics**		
NYHA FC II (%)	72	138
NYHA FC III-IV (%)	28	54
Body mass index (kg/m^2^)	28.1±0.4	183
Abdominal perimeter (cm)	103.0±1.1	158
Heart rate (beats/min)	72±1	190
Systolic blood pressure (mmHg)	117.2±1.6	184
Diastolic blood pressure (mmHg)	72.0±0.9	183
Pulse pressure (mmHg)	45.5±1.4	183
6-min walk distance (m)	408.5±8.0	171

**Symptoms and signs**		
Paroxysmal nocturnal dyspnea (%)	17	30
Reduction exercise tolerance (%)	44	74
Orthopnea (%)	29	51
Syncope (%)	13	23
Lower extremity edema (%)	14	24
Congestion signs (%)	10	10
Jugular venous distension (%)	12	12
Hepatojugular reflux (%)	19	18

**ECG parameters**		
Sinusal rhythm (%)	59	110
Atrial fibrillation (%)	9	17
Necrosis Q waves (%)	32	39
Intervent. Conduct. disorders (%)	63	105
Left bundle branch block (%)	23	26
Pacemaker (%)	55	106
Resynchronization therapy (%)	12	23
QRS length (ms)	134.7±2.8	180
Interval PR (ms)	168.9±3.4	123

**Echocardiographic parameters**		
LVESD (mm)	53.6±0.9	167
LVEDD (mm)	67.4±0.7	181
LVEF (%)	27.3±0.5	192
LAD (mm)	48.2±0.7	173
IVST (mm)	10.4±0.1	180
LVPWT (mm)	10.0±0.1	174
LVH (%)	45	81

**Laboratory values**		
Serum creatinine (mg/dL)	1.18±0.03	189
GFR (mL/min)	56.6±0.7	178
Sodium (mEq/L)	139.8±0.2	190
Potassium (mEq/L)	4.6±0.04	188
Aspartate aminotransferase (UI/L)	26.1±1.4	182
Alanine aminotransferase (U/L)	27.5±2.3	188
Bilirubin (mg/dL)	0.82±0.04	185
Uric acid (mg/dL)	6.98±0.17	132
Glucose (mg/dL)	115.0±2.6	189
Total Cholesterol (mg/dL)	169.4±2.8	172
HDL Cholesterol (mg/dL)	40.7±0.7	167
LDL Cholesterol (mg/dL)	103.3±2.3	167
Triglycerides (mg/dL)	128.7±4.8	180
C Reactive Protein (mg/dL)	0.88±0.19	143
Thyrotropin (mUI/L)	3.1±0.6	171
Thyroxine (ng/dL)	1.31±0.02	150
Hemoglobin (g/L)	136.7±1.2	187
Hematocrit (L/L)	0.419±0.003	189
Erythrocyte count (10E12/L)	4.57±0.04	183
Lymphocytes count (10E9/L)	1.8±0.05	188
Platelet count (10E9/L)	218.2±4.6	184

**Pharmacological treatment**		
ACEI (%)	68	125
ARB(%)	24	43
ACEI and/or ARB (%)	90	168
Beta-blocker (%)	94	176
Ca-antagonists (%)	8	15
Antithromb and/or anticoagul (%)	81	151
Statins (%)	62	116
Antidiabetics (%)	28	52
Diuretics (%)	79	146
Antialdosteronic agents (%)	55	102
Digoxin (%)	11	21
Antiarrhythmics (%)	22	41
Nitrates (%)	13	25
Hydralazine (%)	4	8
Anemia treatment (%)	4	8

NYHA FC (New York Heart Assotiation functional class), AMI (acute myocardial infarction), Intervent conduct disorders (Interventricular conduction disorders), LVESD (left ventricle end-systolic diameter), LVEDD (left ventricle end-diastolic diameter), LVEF (left ventricle ejection fraction), LAD (Left atrial diameter), IVST (Interventricular septum thickness), LVPWT (Left ventricular posterior wall thickness), LVH (Left Ventricular Hypertrophy defined as IVST ≥11 mm),GFR (Glomerular filtration rate), ACEI (Angiotensin Converting Enzyme Inhibitor), ARB (Angiotensin Receptor Blocker), Antithromb and/or anticoagul (Antithrombotic and/or anticoagulant).

**Table 2:** Quartile values of the serum biomarkers studied.

Quartile		Hs-TnT (pg/mL)	Gal-3 (ng/mL)	CICP (ng/mL)	sAXL (ng/mL)	BNP (pg/mL)
1st	11.0		4.9	68.6	69.1	76.2
2nd	17.4		5.8	85.6	82.4	167.7
3rd	28.7		7.5	112.3	98.1	362.7

High sensitivity Troponin T (hs-TnT), galectin-3 (Gal-3), C-terminal propeptide of type I procollagen (CICP), soluble AXL (sAXL) and Brain Natriuretic Peptide (BNP).

**Table 3:** Profiles of HF patients that have a serum biomarker value above the third quartile value.

Characteristic		3Q_Hs-TnT	3Q_Gal-3	3Q_CICP	3Q_BNP
*n*	47		47	45	41
Age (years)	62±1		65±2	58±2	61±2
Sex (%M / %F)	89/11		79/21	80/20	88/12
Hypertension (%)	77		85	58	66
Diabetes mellitus (%)	38		47	27	27
Dyslipidemia (%)	60		57	51	46
Idiopathic etiology (%)	28		34	29	29
Ischemic etiology (%)	51		47	47	46
Valvular etiology (%)	13		11	11	12
Hypertensive etiology (%)	0		2	4	2
Other etiology (%)	8		6	9	10
NYHA FC II (%)/ FCIII_IV (%)	57/43		57/43	56/44	51/49
LVEF (%)	24.2±1.1		26.1±1.1	25.7±1.1	21.4±1.0

High sensitivity Troponin T (hs-TnT), galectin-3 (Gal-3), C-terminal propeptide of type I procollagen (CICP), soluble AXL (sAXL) and Brain Natriuretic Peptide (BNP). New York Heart Association Funtional class II or III_IV (NYHA FC II FCIII_IV), left ventricular ejection fraction (LVEF).

**Table 4:** Pearson correlation analysis of the HF_REF patients’ biomarker levels.

	Number of pairs	*R* coefficient	*P* value
Ln(hs-TnT) vs Ln(Gal-3)	189	0.25	<0.001
Ln(hs-TnT) vs Ln(CICP)	179	0.12	NS
Ln(hs-TnT) vs Ln(sAXL)	191	0.28	<0.0001
Ln(hs-TnT) vs Ln(BNP)	166	0.51	<0.0001
Ln(Gal-3) vs Ln(CICP)	178	0.044	NS
Ln(Gal-3) vs Ln(sAXL)	190	0.27	<0.001
Ln(Gal-3) vs Ln(BNP)	166	0.27	<0.001
Ln(CICP) vs Ln(sAXL)	180	0.17	<0.05
Ln(CICP) vs Ln(BNP)	157	0.26	0.001

**Table 5:** Pearson correlation analysis of the HF_REF patients’ biomarker levels with patients’ biochemical parameters.

Laboratory values	Ln(hs-TnT)			Ln(Gal-3)			Ln(CICP)		
	*N*	*R*	*P*	*N*	*R*	*P*	*N*	*R*	*P*
Serum creatinine(mg/dL)	188	0.43	<0.0001	187	0.34	<0.0001	177	0.15	<0.05
GFR (mL/min)	177	−0.41	<0.0001	177	−0.34	<0.0001			NS
Sodium (mEq/L)	189	−0.24	<0.001	188	−0.21	<0.01			NS
Potassium (mEq/L)			NS			NS			NS
AST (IU/L)			NS			NS	170	0.18	<0.05
ALT (IU/L)			NS			NS			NS
Total bilirubin (mg/dL)	184	0.22	<0.01			NS			NS
Glucose (mg/dL)			NS			NS			NS
Uric acid (mg/dL)	131	0.39	<0.0001	132	0.2	<0.05			NS

GFR (glomerular filtration rate), AST (aspartate transaminase), ALT (alanine transaminase).

**Table 6:** Pearson correlation analyses of the Ln of the biomarkers levels with patients’ blood count and inflammation parameters.

Laboratory values	Ln(hs-TnT)			Ln(Gal-3)			Ln(CICP)		
	*N*	*R*	*P*	*N*	*R*	*P*	*N*	*R*	*P*
Leukocyte count (x10^9^/L)			NS			NS			NS
Platelet count (x10^9^ /L)	183	−0.22	<0.01			NS			NS
Neutrophils (x10^9^/L)	187	0.17	<0.05			NS			NS
Lymphocytes (x10^9^/L)	187	−0.26	<0.001			NS			NS
Monocytes (x10^9^/L)			NS			NS			NS
CRP (mg/dL)	142	0.24	<0.01			NS			NS
Erythrocyte count (x10^12^/L)	182	−0.33	<0.0001			NS			NS
Hemoglobin (g/dL)	186	−0.31	<0.0001			NS			NS
Hematocrit (L/L)	188	−0.27	<0.001			NS			NS

CRP (C-reactive protein).
