# Supplementary material for: Data on clinical characteristics of a heart failure patients’ cohort with reduced ejection fraction and analysis of the circulating values of five different heart failure biomarkers; high sensitivity troponin T, galectin-3, C-terminal propeptide of type I procollagen, soluble AXL and BNP
Source: Data Brief. 2016 Nov 3;9:876–82. doi: 10.1016/j.dib.2016.10.020 (PMC5109253; doi:10.1016/j.dib.2016.10.020)
Supplement: Supplementary file 1 — Supplementary material [file mmc1.docx]

**Conflict of interest**

*Data article*

Data on clinical characteristics of a heart failure patients’ cohort with reduced ejection fraction and analysis of the circulating values of five different heart failure biomarkers; high sensitivity troponin T, galectin-3, C-terminal propeptide of type I procollagen, soluble AXL and BNP.

List of all Authors: M. Batlle, B. Campos, M. Farrero, M. Cardona, B. González, M.A. Castel, J. Ortiz, E. Roig, M.J. Pulgarín, J. Ramírez, J.L. Bedini, M. Sabaté, P. García de Frutos, F. Pérez-Villa

Corresponding Author: Dr. M. Batlle

M. Batlle and P. García de Frutos are inventors on a patent filed for use of sAxl for diagnosis/prognosis of heart failure syndrome (E.U. patent number EP13703603.4). Other authors declare no competing interests.
